# The complete mitochondrial genome of *Xenopsylla cheopis* (Siphonaptera: Pulicidae)

**DOI:** 10.1080/23802359.2021.2017368

**Published:** 2022-01-05

**Authors:** Furong Wei, Xiaokai Jia, Ying Wang, Yuetao Yang, Junyun Wang, Chunhua Gao, Yong Wang

**Affiliations:** a National Institute of Parasitic Diseases, Chinese Center for Disease Control and Prevention (Chinese Center for Tropical Diseases Research), NHC Key Laboratory of Parasite and Vector Biology, WHO Collaborating Centre for Tropical Diseases, National Center for International Research on Tropical Diseases, Shanghai, China; b Department of Forensic Science, School of Basic Medical Sciences, Central South University, Changsha, China

**Keywords:** Mitochondrial genome, *Xenopsylla cheopis*, phylogenetic analysis

## Abstract

*Xenopsylla cheopis*, also called oriental rat flea, is an ectoparasite as well as disease vector for murine typhus and bubonic plague. In the study, the whole mitochondrial genome of *X. cheopis* was sequenced and assembled, which is the second report of mitochondrial genome in the family Pulicidae and the sixth mitochondrial genome in the order Siphonaptera (fleas). The mitochondrial genome is 18,902 bp in length, consisting of 40% A, 44% T, 6% G, and 10% C. Phylogenetic analysis of all available mitochondrial genomes from Siphonaptera indicated that *X. cheopis* clustered with *Ctenocephalides felis* since both species belonged to the family Pulicidae. The complete mitochondrial genome of *X. cheopis* could serve as useful genetic data for investigating the genetic relationship of fleas.

*Xenopsylla cheopis* (Rothschild, 1903) (Siphonaptera: Pulicidae), common name of oriental rat flea, is an ectoparasite as well as disease vector. *X. cheopis* is typically brown color and grows to be approximately 2.5 mm in length. The morphological features of *X. cheopis* are different from cat fleas (*Ctenocephalides felis*) and dog fleas (*Ctenocephalides canis*) by lacking of a pronotal comb, genal comb or divided mesopleuron (Wells and Elston 2020).

The hosts of *X. cheopis* are mammals, primarily Rattus species, such as *Rattus tanezumi* and *Rattus norvegicus* (Xie 1981). Human is an incidental host of *X. cheopis*. If *X. cheopis* leaves the host, it can survive up to six weeks before migrating to a new host (Slavicek 2008). The host switching among sympatric species increases the chance of disease transmission. *X. cheopis* transmits pathogens including *Rickettsia typhi* and *Yersinia pestis*, which can cause murine typhus and bubonic plague, respectively. *R. typhi* in the feces of fleas might enter human body by aerosol inhalation, and infect mainly endothelial cells thought the body causing interstitial pneumonia, meningoencephalitis, fever, etc. Plague is the most severe disease spread by *X. cheopis* that can transmit plague pathogen *Y. pestis* to a new host after feeding on a previous host with bacteremia. After establish infection, *Y. pestis* survives in the gut of fleas and might return to the bite site as the blood meal regurgitates, which could lead to an alternative mode of transmission (Bacot and Martin 1914). The flea-borne transmission of *Y. pestis* to human can be achieved by fleabite. Besides pathogen transmission, the bite of *X. cheopis* itself causes pruritic lesions on human skin. As global warming situation continues, increased population of *X. cheopis* could be an emerging public health issue (Wells and Elston 2020).

The understanding of *X. cheopis* mitochondrial genome (mitogenome) could enrich our knowledge on phylogenetic relation of species from the order Siphonaptera (fleas) that comprises 246 genera (Lewis 1998). However, there are only six mitogenomes from Siphonaptera in GenBank. In the study, the whole mitogenome of *X. cheopis* was sequenced, assembled and annotated, which was the second mitogenome reported in the family Pulicidae.

The *X. cheopis* specimens used in this study were isolated from a trapped house rat (*R. tanezumi*) in Shanghai, China (N31°13′, E121°29′). Collected specimens were identified morphologically and stored in alcohol at 4 °C. A specimen was deposited at National Institute of Parasitic Diseases (en.ipd.org.cn, Contact: Chunhua Gao, email: gaoch@nipd.chinacdc.cn) under the voucher number IPD2020XC01. The total DNA of each rat flea was extracted using QIANamp Micro DNA Kit (Qiangen, Germantown, MD, USA). The sequencing of *X. cheopis* mitogenome was performed on an Illumina HiSeq 2500 system with a paired-end 150 bp sequencing strategy. The mitogenome was assembled using MitoZ v2.3 (Meng et al. 2019). Gene boundaries were identified by MITOS2 Web Server (Bernt et al. 2013). The protein-coding genes (PCGs) and rRNA genes were verified through homology comparison with those of *C. felis*. The tRNA genes were verified using tRNAscan-SE version 2.0 (Chan and Lowe 2019).

The mitogenome of *X. cheopis* is 18,902 bp in length (GenBank accession No. MW310242), consisting of 40% A, 44% T, 6% G and 10% C, which contains 13 PCGs, 22 tRNA genes, 2 rRNA genes, and an AT-rich control region. The gene arrangement follows the typical order of metazoan gene set (Cameron 2014). Most of the PCGs utilize ATA as their start codon except *cox3*, *nad1*, *nad4*, and *nad5* which use ATG. All PCGs ended with a TAA stop codon. For *cox1*, *cox2*, *nad3*, *nad4*, *nad4l*, and *nad5*, TAA stop codon is completed by the addition of 3′ A to the mRNA.

The mitogenome of *X. cheopis* is most closed to that of *C. felis* by comparison with available mitogenomes (Driscoll et al. 2020). Both *C. felis* and *X. cheopis* are under the family Pulicidae. Based on the sequences of 13 PCGs in mitogenome, a phylogenetic tree was constructed using maximum likelihood (ML) method through MEGA X using GTR G + I substitution model (Kumar et al. 2018). Siphonaptera was genetically closed to Mecoptera (scorpionflies), both of which belong to Superorder Endopterygota (Holometabola) (Whiting 2002). Therefore, in the phylogenetic analysis of Siphonaptera, the species from Mecoptera were set as outgroups (Figure 1).

**Figure 1.**
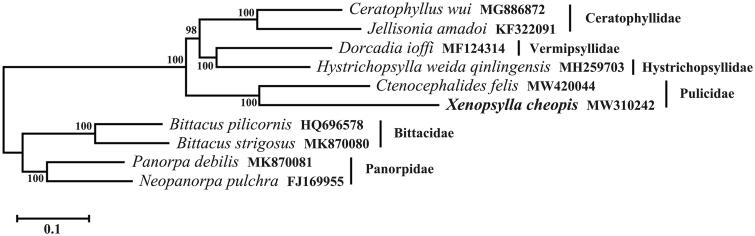
The maximum likelihood tree of six species from Siphonaptera is based on 13 protein-coding genes in mitochondrial genomes. Bootstrap values in percentage are shown at nodes. Four species from Mecoptera were set as outgroups. GenBank accession numbers are listed following species name.

In the phylogenetic tree, *X. cheopis* and *C. felis* clustered together, forming the branch of family Pulicidae. *Ceratophyllus wui* and *Jellisonia amadoi* belonging to the family Ceratophyllidae formed one cluster. *Bittacus strigosus*, *Bittacus pilicornis*, *Panorpa debilis*, and *Neopanorpa pulchra* which belong to the order Mecoptera separated clearly from the species of Siphonaptera. The branches were well supported. In conclusion, the complete mitochondrial genome of *X. cheopis* provided useful genetic data for investigating the genetic relationship of fleas.

## Data Availability

The data that support the findings of this study are openly available in GenBank of NCBI at https://www.ncbi.nlm.nih.gov (GenBank: MW310242 BioProject: PRJNA754093, BioSample: SAMN20741547, SRA: SRR15425125).
